# Supplementary material for: Evaluation of Miscanthus × giganteus Tolerance to Trace Element Stress: Field Experiment with Soils Possessing Gradient Cd, Pb, and Zn Concentrations
Source: Plants (Basel). 2023 Apr 5;12(7):1560. doi: 10.3390/plants12071560 (PMC10096734; doi:10.3390/plants12071560)
Supplement: Supplementary file 1 [file plants-12-01560-s001.zip › plants-2274015-supplementary.pdf]

plants

MDPI

Supplementary materials

Evaluation of *Miscanthus* × *giganteus* tolerance to trace elements stress: Field experiment with soils possessing gradient Cd, Pb and Zn concentrations

Giulia Bastia[1], Karim Suhail Al Souki[2], Bertrand Pourrut[3,*]

This supplementary material was prepared to add the readers more details, for which there was not enough space in the main manuscript, about:

Four experimental plots representing a gradient T.E. concentration were planted with *Miscanthus x giganteus*. Three of which are located in the "Metaleurop" site.

1. Description of the plots

In 2007, two contaminated plots (named M200 and M500) and a control uncontaminated plot (MC) we planted. The MC plot (1.3 ha) is located in a rural area, in Linzeux, about 75 km from the source of contamination. Plot M200 (1.1 ha) is on the workshop site furthest from the source of contamination, approximately 1.8 km from the former foundry, and also the least contaminated. It is located in a peri-urban area, in the town of Courcelles-lès-Lens. Plot M500 (0.8 ha) is located in a peri-urban area, in Evin-Malmaison at a distance of about 1.4 km from the former Pb smelter (Figures S1 and S2).

2. Origin of miscanthus rhizomes and cultural management

On plots M200, M500 and MC, Miscanthus rhizomes from the Bical (currently NovaBiom) company were planted using a potato planter with a density of 20000 plant ha^{-1}. None of these plots were subject to any mineral fertilization.

3. Introducing the M700 experimental plot

In the spring of 2010, the system was reinforced with a new heavily contaminated plot (M700). This plot (0.8 ha) is located north of a wooded experimental site, 1 km from the former industrial site (Figures S1 and S2).

3.1. Origin of Miscanthus rhizomes / plants and cultural management

The aim of introducing M700 pot was to study the influence of different agronomic practices on the T.E. accumulation in different miscanthus organs as well as the yield and biomass produced. The studied agronomic practices were:

- Origin or genotype of Miscanthus
- Planting density: 15000 and 20000 plants ha^{-1}
- Mycorrhization by SolRize® (Agrauxine, Saint Evarzec, France), a strain of endomycorrhizal fungi Glomus sp.

- Nitrogen fertilization in the 3rd year.

The Miscanthus rhizomes used have three distinct origins, referenced "Bical", "Austrian" or "INRA". The rhizomes named “Bical” come from plants taken from the MC plot planted in the spring of 2007 using rhizomes provided on that date by the Bical (currently NovaBiom) company. The rhizomes referenced “Austrian” were provided by the company Rhizosfer whose head office is in Brienne sur Aisne. The rhizomes under the name "INRA" come from the farm that supplied Isabelle Lamy as part of the RESACOR program.

3.2. M700 plot description

In order to determine the influence of origin, planting density, addition of an endomycorrhizal inoculum and fertilization, 24 modalities were studied on the plot. Each modality was repeated three times. In total, 72 subblocks (4 m x 10 m) were installed in the plot. They were spread over six blocks separated by a strip 5 m wide (Figure S3).

The plots with the addition of the endomycorrhizal inoculum SolRize® were grouped together in the lower part of the plot in order to limit any spread, by superficial runoff of rainwater, of mycorrhizae towards the plantations that have not been inoculated. The distribution of modalities and repetitions in the inoculated or uninoculated blocks was carried out randomly by drawing lots.

Each plot has six rows of Miscanthus with a length of 10 m. The planting density was obtained by varying the distance between the plants on the rows. Planting density was 15000 or 20000 plants ha^{-1}.

In July 2011, the paths were grassed by the Chamber of Agriculture. From May to October 2011, the MV plot was subjected to weeding.

In June 2012, 50 units of nitrogen, in the form of ammonium nitrate (27%), were added manually to half of the system (i.e. 36 plots). This fertilization aimed to compensate for exports and strengthen the rhizomatous system.

Finally, it is noteworthy to mention that in the current work, the studied miscanthus samples from the M700 experimental plot were of Bical cultivar (Mis-B), collected from the subplots with plantation density 20000 plant ha^{-1}. Moreover, these subplots were not subjected neither to fertilization nor to mycorrhization.

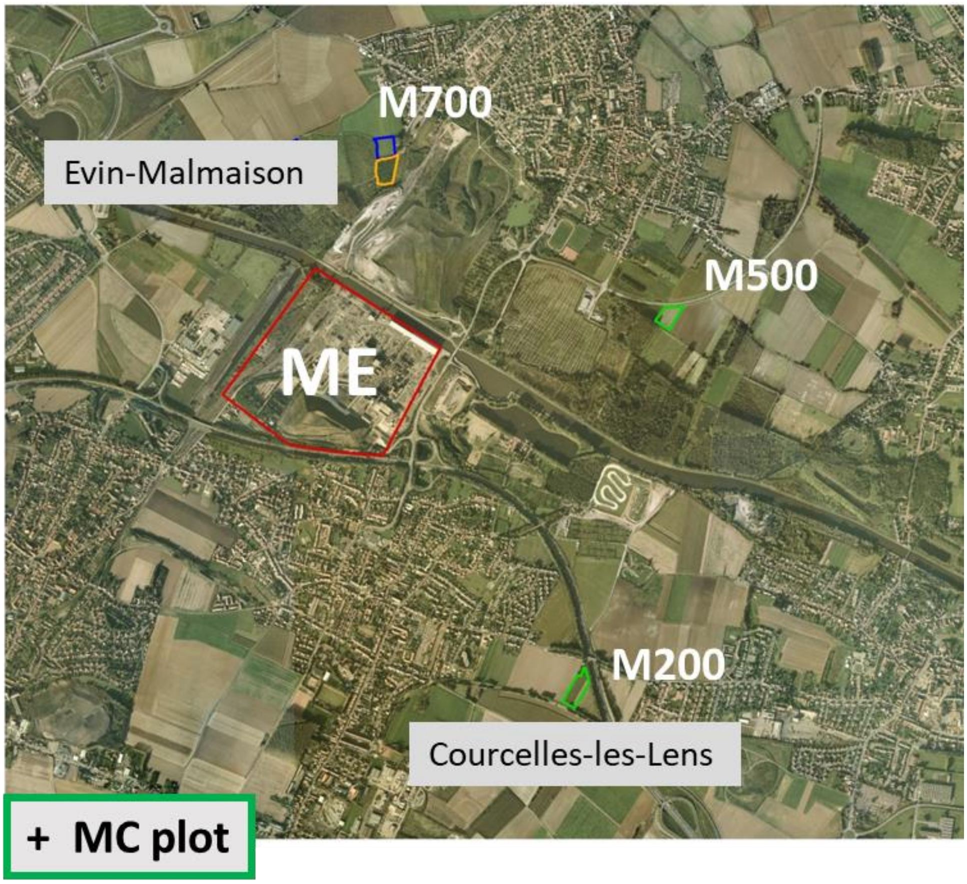

Figure S1: Location of T.E. contaminated experimental agricultural plots (M200, M500 and M700) near the former Pb smelter (Metaleurop Nord, ME) in Northern France. MC plot is the uncontaminated control plot.

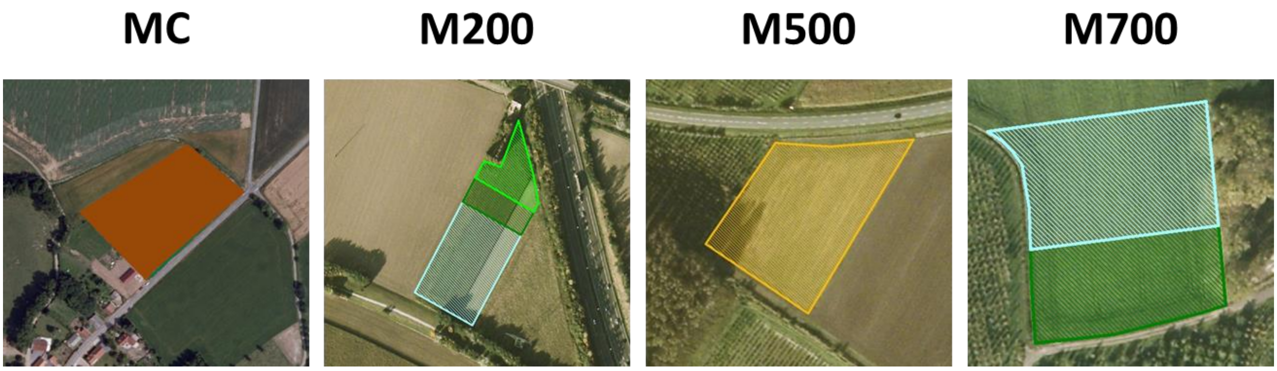

Figure S2: Aerial image and delineation MC, M200, M500 and M700 experimental plots.

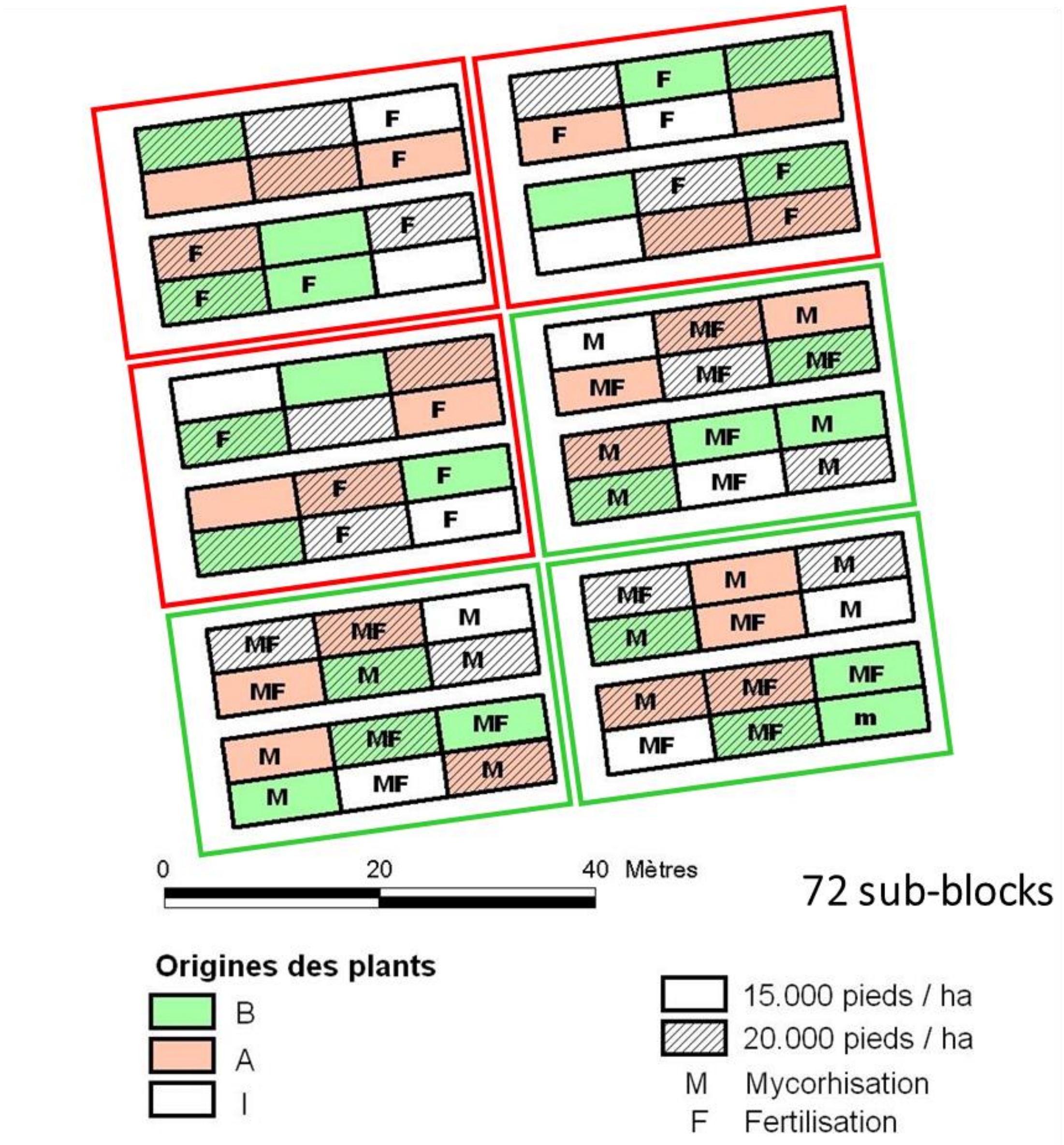

Figure S3: Detailed map of the different agronomic practices performed in M700 plot.
1 block:

- 12 randomized subblocks
- 3 Miscanthus cultivars/origins (B, A and I)
- 2 plantation densities (15000 and 20000 plants ha^{-1})

3 blocks (replicates) without mycorrhization and 3 blocks with mycorrhization.

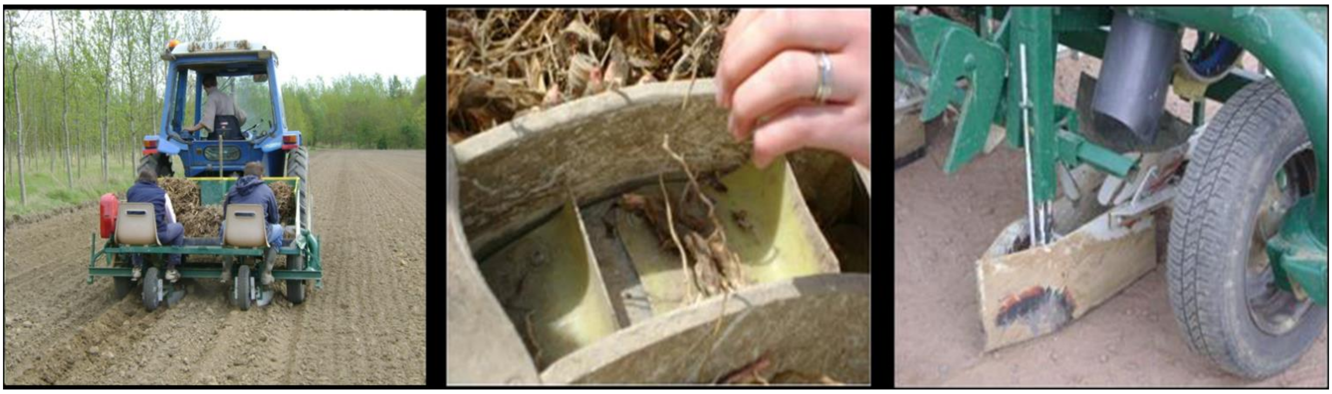

Figure S4: Mechanized mode of planting rhizomes in MC, M200, M500 and 700 plots

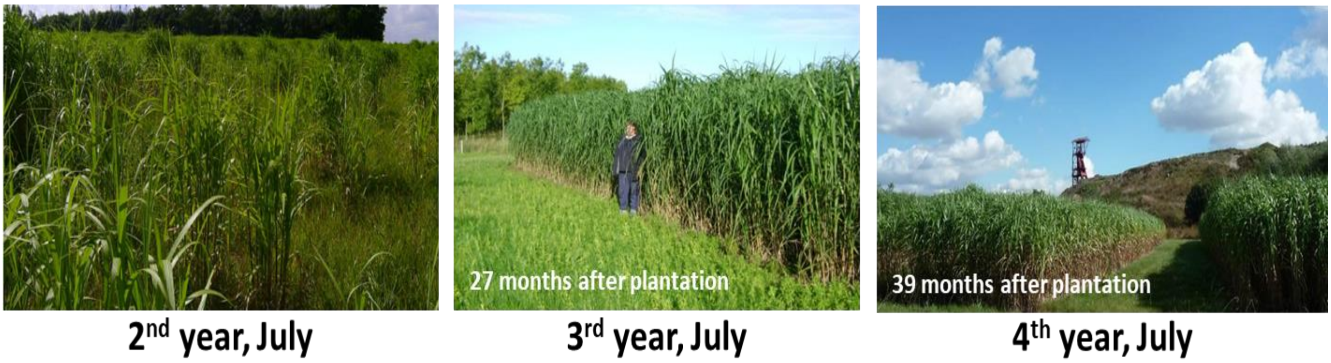

Figure S5: Evolution of miscanthus growth in the most contaminated M700 plot
